# Supplementary material for: Cohort profile of a prospective cohort study among middle-aged community-dwellers in rural Vietnam: The Khánh Hòa Cardiovascular Study
Source: PLoS One. 2024 Dec 3;19(12):e0312525. doi: 10.1371/journal.pone.0312525 (PMC11614239; doi:10.1371/journal.pone.0312525)
Supplement: S1 Table — (DOCX) [file pone.0312525.s001.docx]

**S1 Table. Specific Criteria Used to Define Health Outcomes in the Khánh Hòa Cardiovascular Study, Vietnam**

| **Health outcomes** | **Experimental/measurement conditions** | **Definitions** | **References** |
| --- | --- | --- | --- |
| **Excess body weight** | Height and weight were measured without shoes and wearing light clothes to the nearest 0.1 kg and 0.1 cm, using a portable stadiometer (Charder, HM200P, Tokyo, Japan) and a digital scale (Tania, HD-661, Tokyo, Japan), respectively.  BMI was calculated from the measured height and weight (kg/m^2^). | Underweight: BMI <18.5; normal: BMI 18.5–24.9; overweight: BMI 25–29.9; and obesity: BMI ≥30.0 kg/m^2^ | WHO [20] |
| **Waist-to-height ratio** | With the participant standing upright with his/her arms at the sides, the waist circumference was measured at the midpoint between the lowest rib and iliac crest using a tape measure. | Waist-to-height ratio was calculated by dividing waist circumference (cm) by height (cm). | Carmienke et al. [18] and Abdi Dezfouli et al. [19] |
| **Hypertension** | An electric sphygmomanometer (Omron, HEM-1020, Tokyo, Japan) was used to measure blood pressure twice, after a 5-minute rest before the first measurement. Participants sat with their arms supported at the level of their hearts. The two measurements were used to calculate the mean SBP and DBP. | SBP ≥140 mmHg, DBP≥90 mmHg, or the use of antihypertensive medication | WHO [21] |
| **Diabetes^a^** | Plasma fasting glucose was measured using Cobas 8000 (Roche, Basel, Switzerland), and HbA1c was quantified by high-performance liquid chromatography using the HLC-723 G8 system (Tosoh Bioscience, Tokyo, Japan). | Fasting plasma glucose ≥7 mmol/L (≥126 mg/dL), HbA1c ≥6.5% or self-reported use of antidiabetic medication | American Diabetes Association [22] |
| **Prediabetes^a^** | Please see above. | Fasting plasma glucose of 5.9–6.9 mmol/l (100–125 mg/dL) or HbA1c of 5.7–6.4% among those without diabetes | American Diabetes Association [22] |
| **Dyslipidemia^a^** | The blood sample was tested for total cholesterol, LDL-C, HDL-C, and triglyceride using an automated analyzer (Roche, Cobas 8000, Basel, Switzerland). | Total cholesterol ≥240 mg/L, LDL-C ≥160 mg/L, HDL-C <40 mg/L, triglycerides ≥200 mg/L, or the use of anti-dyslipidemia medication. | NCEP-ATP III [23] |
| **Elevated C-reactive protein** | The C-reactive protein concentration was measured using an automated analyzer (Roche, Cobas 8000, Basel, Switzerland). | 3.0–9.9 mg/L (subclinical inflammation); ≥10 mg/L (acute inflammation) | Pearson et al. [24] |
| **Metabolic syndrome** | Please see above. | At least three of the following five conditions:  (1) waist circumference ≥90 and ≥80 cm for men and women, respectively; (2) fasting plasma glucose ≥6.5 mmol/L (100 mg/dL) or receiving antidiabetic medication,  (3) SBP ≥130 mmHg, DBP ≥85 mmHg, or on antihypertension medication,  (4) HDL-C <1.04 mmol/L (40 mg/dL) for men and <129 mmol/L (50 mg/dL) for women,  (5) triglycerides ≥1.7 mmol/L (150 mg/dL) or on lipid-lowering medication. | NCEP-ATP III [23] |
| **Depressive symptoms** | Depressive symptoms were measured by the shortened 11-item version of the Center for Epidemiologic Studies Depression (CES-D) questionnaire, which elicits how often participants had experience symptoms associated with depression (e.g., loss of appetite, sad feelings, loneliness, and low-quality sleep). Each question was scored from 0 to 3, with the following response options: “rarely or none of the time (score = 0),” “some or little of the time (score = 1),” “occasionally or moderate amount of the time (score = 2),” and “most of the time (score = 3).” | A cutoff score ≥9 out of 33 was used to define depressive symptoms. | Kohout et al. [17] |

BMI, body mass index; CES-D, the Center for Epidemiologic Studies Depression (CES-D) questionnaire; DBP, diastolic blood pressure, HbA1c, glycated hemoglobin; HDL-C, high-density lipoprotein cholesterol; LDL-C, low-density lipoprotein cholesterol; SBP, systolic blood pressire; WHO, World Health Organization.

^a^All participants were asked to fast for >8 h prior to blood sampling, with samples obtained by venipuncture (and collected in an NaEDTA tube and a serum tube) that were subsequently centrifuged at 3000 revolutions per minute for 30 minutes at the study sites, and thereafter transported to the Pasteur Institute in Nha Trang at temperatures <4°C.
