# Supplementary material for: Cohort profile of a prospective cohort study among middle-aged community-dwellers in rural Vietnam: The Khánh Hòa Cardiovascular Study
Source: PLoS One. 2024 Dec 3;19(12):e0312525. doi: 10.1371/journal.pone.0312525 (PMC11614239; doi:10.1371/journal.pone.0312525)
Supplement: S2 Table — (DOCX) [file pone.0312525.s002.docx]

**S2 Table.** **Prevalence of Selected Health-related Outcomes Ascertained from the STEP Survey Data**

|  | **Hypertension^a^** | **Excess body weight^b^** | **Smoking^c^** | **Alcohol^d^** |
| --- | --- | --- | --- | --- |
| **Men, age group, years** |  |  |  |  |
| **30–49** | 22.3 (18.4–26.2) | 17.3 (14.0–20.5) | 59.7 (55.5–63.7) | 81.8 (78.5–85.2) |
| **50–69** | 45.5 (39.9–51.0) | 16.1 (12.3–19.9) | 52.2 (47.2–57.2) | 71.2 (66.9–75.6) |
| **40–60** | 35.6 (31.1–40.4) | 17.2 (14.0–20.8) | 61.0 (56.6–65.3) | 79.7 (76.1–82.9) |
| **Women, age group, years** |  |  |  |  |
| **30–49** | 12.3 (9.6–14.9) | 18.0 (14.9–21.1) | 1.4 (0.5–3.6) | 14.5 (11.7–17.3) |
| **50–69** | 35.4 (30.5–40.4) | 22.8 (18.5–27.1) | 2.1 (1.1–3.7) | 6.3 (4.3–8.4) |
| **40–60** | 25.0 (21.7–28.6) | 23.9 (20.4–27.7) | 1.9 (0.9–4.1) | 9.8 (7.6–12.4) |

To estimate the prevalence of each health-related outcome among individuals aged 40–60 years, for which the information was unavailable in the WHO Country Report for the STEPS Study 2015, we used publicly available data from the WHO website (<https://extranet.who.int/ncdsmicrodata/index.php/catalog/590/related-materials>). The original WHO STEPS Survey report estimated the prevalence using proprietary statistical programs based on Epi-Info/Microsoft Access; therefore, we adhered to similar definitions to replicate their approach. The prevalence estimates for individuals aged 30–49 and 50–69 years were directly extracted from the WHO STEP survey report, except for current smoking, which was not presented in the report. We confirmed that the figures are almost identical for both methods.

^a^Hypertension was defined as a mean systolic blood pressure ≥140 mmHg (variables “m5a” and “m6a”), a mean diastolic blood pressure ≥90 mmHg (variables “m5b” and “m6b”), or the self-reported use of antihypertensive medication (variable “m7”). The sampling weight “wstep2” was employed to generate estimates for a representative population.

^b^Excess body weight was defined as a body mass index (BMI) ≥25 kg/m^2^, calculated using variables “m11” (height) and “m12” (weight). The sampling weight 'wstep3' was applied to generate estimates for a representative population.

^c^Current smoking status was determined based on an affirmative response to the question, "Do you currently smoke?" (variable “t1”). The sampling weight “wstep1” was used to generate estimates for a representative population.

^d^Current alcohol consumption was defined as alcohol consumption in the past 30 days, based on responses to questions about alcohol consumption, “a1” (ever drinking), “a2” (drinking in the previous one year), and “a5” (drinking in the previous 30 days). The sampling weight “wstep1” was used to generate estimates for a representative population.
