# Supplementary material for: Correction: Cohort profile of a prospective cohort study among middle-aged community-dwellers in rural Vietnam: The Khánh Hòa cardiovascular study
Source: PLoS One. 2025 Mar 28;20(3):e0321417. doi: 10.1371/journal.pone.0321417 (PMC11952203; doi:10.1371/journal.pone.0321417)
Supplement: S1 File — (PDF) [file pone.0321417.s001.pdf]

PLOS ONE

RESEARCH ARTICLE

Cohort profile of a prospective cohort study among middle-aged community-dwellers in rural Vietnam: The Khánh Hòa cardiovascular study

Chau Que Nguyen[1], **Thuy Thi Phuong Pham**[1], **Danh Cong Phan**[1], **Hung Thai Do**[2], **Tetsuya Mizoue**[3], **Yosuke Inoue**[3*]

1 Department of Non-communicable Disease Control and Nutrition, Pasteur Institute in Nha Trang, Nha Trang, Khánh Hòa, Vietnam, **2** Pasteur Institute in Nha Trang, Nha Trang, Khánh Hòa, Vietnam, **3** Department of Epidemiology and Prevention, National Center for Global Health and Medicine, Tokyo, Japan

* yosuke.yoshi.yosky@gmail.com

Check for updates

OPEN ACCESS

Citation: Nguyen CQ, Pham TTP, Phan DC, Do HT, Mizoue T, Inoue Y (2024) Cohort profile of a prospective cohort study among middle-aged community-dwellers in rural Vietnam: The Khánh Hòa cardiovascular study. PLoS ONE 19(12): e0312525. https://doi.org/10.1371/journal.pone.0312525

Editor: Chinh Quoc Luong, Bach Mai Hospital, VIET NAM

Received: May 28, 2024

Accepted: October 9, 2024

Published: December 3, 2024

Copyright: © 2024 Nguyen et al. This is an open access article distributed under the terms of the Creative Commons Attribution License, which permits unrestricted use, distribution, and reproduction in any medium, provided the original author and source are credited.

Data Availability Statement: The data supporting the findings of this study are not publicly available due to privacy concerns and the potential for participant identification. Given the sensitive nature of the collected data, including detailed personal health information, lifestyle habits, and early-life experiences, combined with the publication of specific study locations, there is a heightened risk of participant identification. This restriction is in accordance with the decisions made by the Research Ethics Committees at the National Center

Abstract

Disease burden associated with cardiovascular diseases (CVDs) in low- and middle-income countries has been on an increasing trend in the past decades. Despite the worldwide genetic, cultural, and environmental variations in determinants of CVDs, few studies have attempted the identification of risk factors of CVDs in low- and middle-income countries. This article aims to introduce the Khánh Hòa Cardiovascular Study, a prospective cohort study among middle-aged community dwellers in rural Khánh Hòa, Vietnam. A total of 3000 individuals, aged 40–60 years at baseline, participated in the baseline survey conducted from June 2019 to June 2020 and will be followed up for the subsequent 10 years. The baseline survey collected information on sociodemographic variables, disease history, lifestyle, social environment, and mental health via questionnaires, physical examinations, and biochemical measurements. Information on the incidence of severe health outcomes (i.e., mortality, CVDs, and cancer) has been and will be collected using a study-specific disease registry. Results showed that the prevalences of excess body weight (body mass index $\geq$25 kg/m^2), hypertension, diabetes mellitus, and dyslipidemia were 25.9%, 39.6%, 10.2%, and 45.1%, respectively. Furthermore, by March 2023, 21 participants had died, including 5 CVD deaths and 12 cancer deaths. Moreover, we recorded 22 and 31 cases of nonfatal CVDs and cancer, respectively. These results suggest that many rural residents in Vietnam have high cardiometabolic risk, and underscore the importance of advancing research to identify risk factors and prevent the onset of serious health events.

Introduction

Cardiovascular diseases (CVD) are the leading cause of mortality worldwide [1,2], and the World Health Organization (WHO) estimates showed for approximately 17.9 million CVD-

for Global Health and Medicine in Japan and the Pasteur Institute in Nha Trang, Vietnam, which have not approved the release of such data publicly. However, the data are available upon reasonable request to the Department of Epidemiology and Prevention at the National Center for Global Health and Medicine (yoboinfo@hosp.ncgm.go.jp) or to the corresponding author (yosuke.yoshi.yosky@gmail.com).

Funding: This work was supported by The National Center for Global Health and Medicine (19A06, 22A02 awarded to YI), The Pfizer Health Research Foundation (awarded to YI), and The Japan Society for the Promotion of Science (KAKANHI) (JP19K20536, JP23H03638 awarded to YI, JP21J01171 awarded to Aki Yazawa, JP21K17301 awarded to Dong Van Hoang). The funders had no role in study design, data collection and analysis, decision to publish, or preparation of the manuscript. Aki Yazawa and Dong Van Hoang did not contribute specifically to this work, but we have used the research grants awarded to them to manage the cohort.

Competing interests: The authors have declared that no competing interests exist.

related deaths, which comprised 31% of all global deaths, in 2019 [1]. As an attempt to mitigate the CVD-associated disease burden, numerous epidemiological studies investigated risk factors of CVD, including behavioral risk factors (e.g., smoking, physical inactivity, and unhealthy diet), clinical risk factors (e.g., hypertension, diabetes, and dyslipidemia) [2,3], and social determinants [4].

In contrast to high-income countries (HIC) that have reported robust evidence, low- and middle-income countries (LMIC) have reported limited epidemiological evidence, possibly because of insufficient research-related human capacity and funding [5]. Given the genetic, cultural, and environmental variations in the determinants of CVD that exist worldwide, this underrepresentation of LMICs in CVD research has resulted in an incomplete understanding of the epidemiology of CVD [6]. Moreover, compared to HICs, LMICs have been more disproportionately burdened with CVDs and account for more than 80% of all CVD deaths [7]. Thus, effective strategies to prevent and reduce the CVD-associated disease burden in LMICs will help improve global health.

Vietnam has experienced rapid economic growth since the introduction of a market-oriented policy in 1986 [8], and the gross domestic product per capita has been estimated to increase from USD706 to USD4320 during the period between 1986 and 2023 [9]. Although this economic growth has extended life expectancy at birth from 69.09 years, in 1986, to 75.77 years, in 2023 [10], the prevalence and incidence of noncommunicable diseases (NCD), including CVD, has also increased. Cerebrovascular and ischemic heart disease are the leading causes of premature mortality in Vietnam [11]. Despite this, epidemiological data remain scarce, and we are not aware of any prospective cohort study that has investigated these diseases in Vietnam.

This article aims to describe the Khánh Hòa Cardiovascular Study (KHCS), in which we aim to identify CVD determinants in the Vietnamese context, to enable better-informed strategic planning to mitigate the CVD-associated disease burden and the related morbidity and mortality in the country. This article provides an overview of the collected data and summarizes the main results published to date.

Methods

Cohort, study design, and study population

The KHCS is a collaborative effort of the Pasteur Institute in Nha Trang (PINT), Vietnam, a national research institute under the Vietnamese Ministry of Health with expertise in infectious diseases, epidemiology, and vaccine development (http://www.pasteur-nhatrang.org.vn/en/), and the National Center for Global Health and Medicine, Japan (NCGM), known for research on infectious and immunological diseases, diabetes, and metabolic diseases as well as advanced medical care, international medical collaboration, and training initiatives (https://www.ncgm.go.jp/en/index.html). Initially focused on infectious diseases, this collaboration has expanded since 2018 to include research and initiatives on NCDs in response to the escalating CVD- and NCD-associated disease burden in Vietnam.

The KHCS was designed as a prospective cohort study and was conducted in the Cam Lâm district, Khánh Hòa Province, located on the south-central coast of Vietnam (Fig 1); the study sites are in a suburban area, which is situated approximately 35 km from Nha Trang, the provincial capital. The primary industries in the district include agriculture, aquaculture, forestry, and manufacturing. The region has a tropical savanna climate, with annual average temperature and precipitation of 26.5˚C and 1768 mm, respectively [12].

We selected eight of 14 communes in the district because of their proximity to Nha Trang with the aim of following up the study cohort for an extended period. Participants aged 40 to

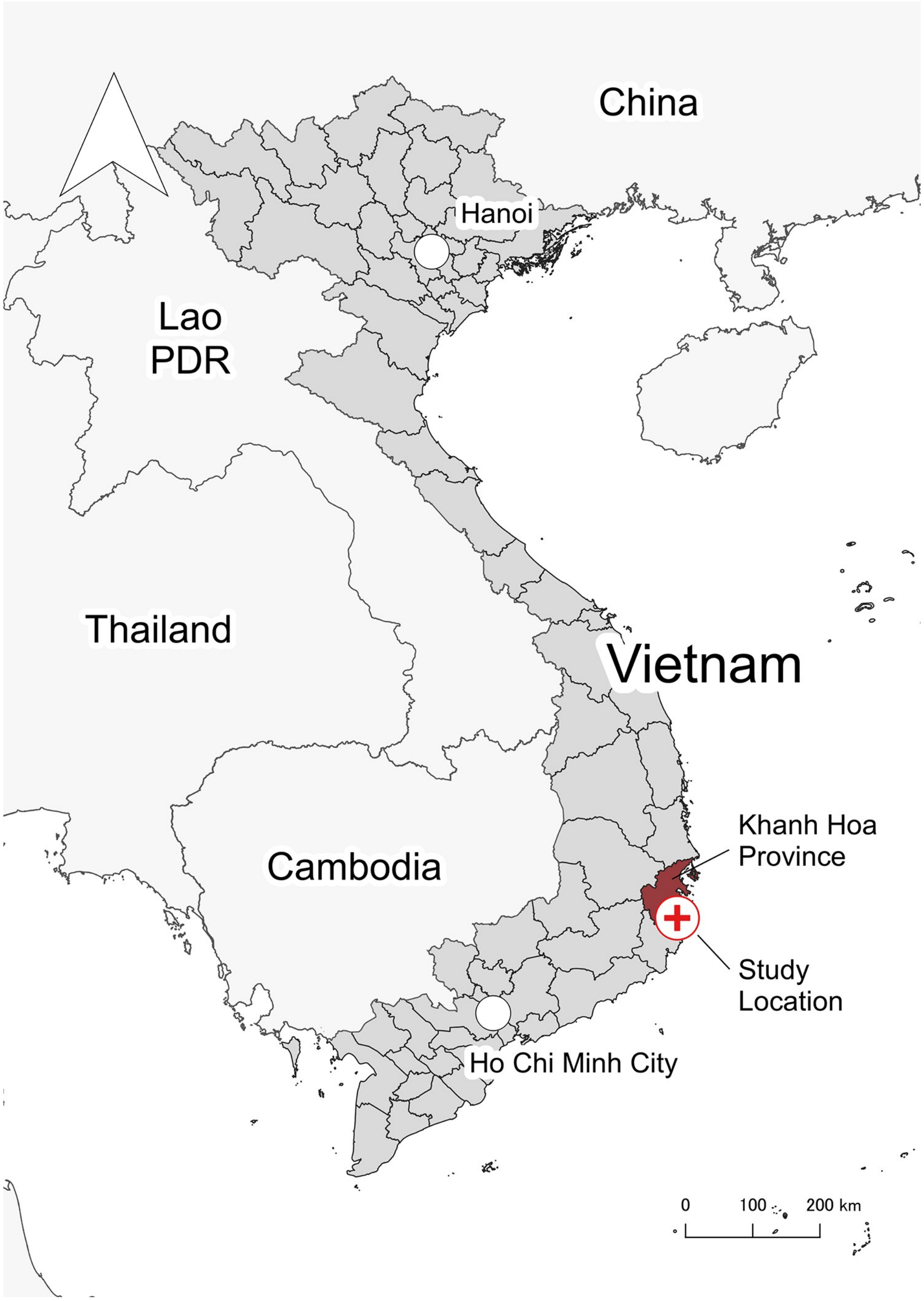

Fig 1. The map of the study location. Source of the GeoJSON files: Vietnam: https://data.opendevelopmentmekong.net/dataset/vietnam-province-iso-codes; Cambodia: https://data.opendevelopmentcambodia.net/dataset/administrative-boundaries-of-cambodia-2014; Laos: https://data.laos.opendevelopmentmekong.net/dataset/laos-administrative-boundaries-country-province-and-district; Thailand: https://data.humdata.org/dataset/cod-ab-tha; and China: https://data.humdata.org/dataset/geoboundaries-admin-boundaries-for-china. The files for Vietnam, Cambodia, and Laos are licensed under the CC BY-SA 4.0, the file for Thailand is licensed under the CC-BY-IGO, and the file for China is licensed under the CC BY 4.0 and the ODC-ODbL.

https://doi.org/10.1371/journal.pone.0312525.g001

Table 1. Selection of Study Participants in the Baseline Survey of Khánh Hòa Cardiovascular Study, Vietnam (2019–2020).

	Commune								Pilot Survey[b]	Total
	A	B	C	D	E	F	G	H		
**Villages[a]**	3	3	16	8	7	4	4	8		
**Selected villages**	3	2	9	1	4	2	2	2		
**Individuals aged 40–59 years in the residents' registry in the selected villages**	913	837	1283	677	913	1543	950	970	-	-
**Candidate participants listed initially by the Commune Health Centers[c]**	469	523	428	366	449	469	474	419	-	-
**Participants, n**	381	387	317	328	393	374	400	353	67	3000
**Population coverage rate (%)[d]**	41.7	46.2	24.7	48.4	43.0	24.2	42.1	36.4	-	-

[a] A village is a residential unit within a commune.

[b] The pilot survey, conducted in June 2019, included 100 participants aged 30–59 from the eight villages in Commune D. In the main survey, we used data from 81 individuals aged 40–59, with 14 participants from the village where we recruited for the main survey (the number is included in the column for Commune D) and the remaining 67 from the other seven villages.

[c] Candidate participants listed initially by the Community Health Centers (CHCs) were those identified by CHCs as residents who could participate in the study.

[d] The population coverage rate was calculated as the number of participants divided by the number of registered individuals aged 40–59 years, regardless of the eligibility criteria.

https://doi.org/10.1371/journal.pone.0312525.t001

59 years were chosen to focus on a population at higher risk for developing CVD, which tends to increase in incidence during middle age. This approach ensures a sufficient number of cases during the observation period, enabling a more robust analysis of associated risk factors. Furthermore, by examining a wide range of CVD risk factors during middle age rather than later in life, we aim to identify risk factors that are essential for early interventions to best minimize the disease burden associated with CVD.

Each commune health center aimed to recruit approximately 400 participants, continuing recruitment efforts until the sample size reached 3,000 (i.e., convenience sampling) (Table 1). The exclusion criteria were as follows: institutionalized individuals, those who were unable to provide informed consent, those who had a plan to move out of the community within 1 year, pregnant women or those who gave birth within 1 year, and those who had a history CVD events.

The specific recruitment procedure involved the following steps: Commune health centers have access to the residents' registry, which is regularly updated by the local government, and this enabled the initial identification of households with residents aged 40–59 years. Staff members from these commune health centers selected a village (i.e., a residential unit within a commune) and visited each household to verify the eligibility and willingness of individuals in this age group to participate in the survey. If they did not recruit a sufficient number of candidates, they selected another village and repeated the process until the required number of candidate participants was attained. Candidate participants were approached with invitation letters delivered in person to participate in the survey until the target sample size of 3000 was reached.

All participants provided written informed consent before study participation and were informed that they could withdraw their consent at any time. The research ethics committees of the NCGM (approval number: NCGM-G-003172-03) and the PINT (02/2019/HDDD-IPN) approved this study.

Baseline survey

The baseline survey commenced, in June 2019, with a pilot phase, during which the number of participants was limited to 25 per day for training purposes; the main survey was conducted

from November 2019 to June 2020. Participants were instructed to visit the commune health center corresponding to their place of residence during the morning hours, after fasting for at least 8 hours before the sample collection. Individuals who did not adhere to the fasting protocol, specifically by consuming breakfast, were instructed to return on different days to ensure compliance with the essential fasting requirements for the study. Anthropometry, blood samples for biochemical measurements, and sociodemographic and lifestyle parameters were collected during face-to-face interviews, as described below.

Questionnaire survey. The study questionnaire was developed by referring to the WHO STEP Survey [13], although other questions deemed essential for capturing various health determinants in the Vietnamese context, for a more comprehensive questionnaire, were added. The information collected from the baseline survey is presented in Table 2.

Sociodemographic variables included age, sex, marital status, insurance number, educational attainment, job category, household income, and household assets. Participants were asked to choose one of the response options for education (i.e., did not go to school; less than primary school; primary school; junior high school; high school; university; and attained further education) and job categories (i.e., government employee; non-government employee; self-employed; farmer or fisherman; homemaker; other; and unemployed), respectively. To calculate an equivalized income value, household income per capita was estimated based on

Table 2. Summary of items collected in the baseline survey of the Khánh Hòa cardiovascular study, Vietnam (2019–2020).

Items	Description	Specific instruments
**Sociodemographics**	• Age, sex, ethnicity, and marital status, insurance number<br>•Educational attainment, occupation, household income, and household assets	• Based on the WHO STEP Survey [13]
**Lifestyle**	• Smoking status or passive smoking<br>•Alcohol consumption<br>•Physical activity<br>•Sleep duration	• Based on the WHO STEP Survey [13]
**Diet**	• Vegetable and fruit<br>•Rice, noodles, and bread<br>•Meat consumption, fish consumption, and soy<br>•Milk, yogurt, and cheese<br>•Sweetened beverages, coffee, and tea (green tea and other tea)<br>•Sodium consumption<br>•Frequency of skipping breakfast, eating outside, and eating alone	• Information on vegetable and fruit consumption and sodium consumption were retrieved using questions employed in the WHO STEP Survey [13]<br>•Information on the other food items were collected using food-frequency questionnaires [14,15].
**Social environment**	• Social capital (structural and cognitive social capital)<br>•Childhood socioeconomic status<br>•Parental absence before age 15	• Questions on social capital were retrieved from the Adapted Social Capital Assessment Tool (SASCAT) [16].
**Medical characteristics**	• Disease history and treatment status (hypertension, diabetes, and dyslipidemia)<br>•Family history of cancer and CVD	• Information on disease history and treatment status (hypertension, diabetes, and dyslipidemia) were collected using questions of the WHO STEP Survey [13].
**Physical examination**	• Height, weight, and waist circumference<br>•Blood pressure	
**Biochemical measurement**	• Total cholesterol, high-density lipoprotein cholesterol, low-density lipoprotein cholesterol, and triglyceride<br>•Insulin, glucose, and glycated hemoglobin<br>•Uric acid<br>•High-sensitivity C-reactive protein	
**Mental health**	• Depressive symptoms	• The Center for Epidemiological Scale-Depression [17]

CVD: Cardiovascular diseases; WHO: World Health Organization.

https://doi.org/10.1371/journal.pone.0312525.t002

responses regarding monthly household income and the number of household members. Household assets were assessed by asking about the possession of several items in households (e.g., cars, motorbikes, television, and mobile phones).

Lifestyle-related variables included smoking status, alcohol consumption, physical activity, fruit and vegetable consumption, consumption of other selected food items (e.g., rice, bread, noodles, meat, coffee, and tea), and sleep duration (hours). Questions on smoking status, alcohol consumption, and fruit and vegetable consumption were derived from the STEP survey questionnaire [13]. For other dietary components, we employed the methodology from previous research that used food-frequency questionnaires [14,15]. Specifically, we asked them to provide their consumption frequency (i.e., the number of days per week and the number of times per day) and choose one of three response options for the consumption amount, which were based on a comparison with standard amounts illustrated on the show cards (i.e., smaller, equivalent, and larger). The list of food items was determined after group discussions with the PINT and CHC staff members who were familiar with the local diet.

We asked the participants questions regarding their social environment, including social capital (cognitive and structural social capital) based on a previously validated questionnaire [16], self-reported childhood socioeconomic status with five response options (i.e., high; upper-middle; middle; lower-middle; low), and parental absence in childhood, for which we ascertained the causes (i.e., death, divorce, and parents' out-migration to other locations) and timing (i.e., before 3 years of age, or after 3 years to before 15 years of age).

Information on the history of hypertension, diabetes, dyslipidemia, cancer, and diseases of the circulatory system was collected via a questionnaire. Furthermore, participants were asked about their family history of hypertension, diabetes, dyslipidemia, cancer, and CVD. Depressive symptoms were assessed using the 11-item Center for Epidemiological Scale-Depression [17].

Physical examination and biochemical measurement. Experienced staff members from PINT, who had previously conducted physical measurements in other surveys, used a digital scale (Tania, HD-661, Tokyo, Japan) and a portable stadiometer to measure body height and weight to the nearest 0.1 cm and 0.1 kg, respectively. Measurements were taken with the participants wearing light clothing and without shoes. Body mass index (BMI) was calculated by multiplying the weight (kg) by the height squared (m^2). Waist circumference measurements were taken using a tape measure placed over the light clothing worn by the participants, and the waist-to-height ratio (WHtR) was then calculated by dividing the waist circumference (cm) by height (cm) [18,19].

With the participants seated and their left arm held at heart level, blood pressure was measured twice using an electric sphygmomanometer (Omron, HEM1020, Tokyo, Japan). Prior to the first measurement, participants were instructed to relax in a seated position for at least 5 min, and was increase to 20 min for those who arrived at the venue by bicycle. The mean systolic and diastolic blood pressures were calculated using two measurements. A mean systolic blood pressure $\geq$140 mmHg, a mean diastolic blood pressure $\geq$90 mmHg, or the self-reported use of antihypertensive medication were all considered indicative of hypertension.

Blood samples were collected through venipuncture, centrifuged at the study sites, and transported at temperatures below 4˚C to the PINT laboratory. Fasting blood samples were used to measure insulin, glucose, low-density lipoprotein (LDL) cholesterol, high-density lipoprotein (HDL) cholesterol, triglycerides (TG), and C-reactive protein (CRP) using the Cobas 8000 (Roche, Switzerland), whereas the HbA1c was determined using the HLC-723 G8 high-performance liquid chromatography system (Tosoh Bioscience, Japan). These devices pass annual accuracy inspections that are conducted by the governmental regulatory departments.

Definitions of general obesity [20], hypertension [21], diabetes and prediabetes [22], dyslipidemia and metabolic syndrome [23], and elevated CRP levels [24] were adopted from previous studies and are detailed in S1 Table.

Follow-up of study participants

We conducted follow-ups with the participants at 1- to 2-year intervals, for a maximum duration of 10 years. CHC staff used the participants' identification numbers to search for information on mortality and health outcomes in the electronic databases (*Phần mềm thống kê y tế* for mortality records, and *Ph n mềm khám chữa bệnh BHYT* and *HIS.ONE* for health events). All mortality cases within the area are reported to the Commune People's Committee and documented in the system and in a death register, referred to as "*A6*," at the commune health center. The medical database included records of insured individuals treated at core hospitals in the area. CHC staff members collected data on diagnosed health events from the medical database (e.g., specific disease names and associated ICD codes) without considering specific case definitions, and detailed information on these is presented in Table 3. Subsequently, researchers at the PINT and NCGM extracted health events that matched our case definitions. For individuals without insurance or insurance numbers as well as those whose information was not available in the database, we also conducted telephonic surveys. Household visits were conducted to verify specific documents, such as medical certificates for individuals who self-reported severe health outcomes but lacked the corresponding information in the database. Information on the causes of death and severe health outcomes was coded according to the International Classification of Diseases, 10th Edition (ICD-10), and recorded in a study-specific registry.

We verified the participants' residence in the commune by checking the database, conducting telephonic interviews, and conducting household visits. Additionally, CHC staff, who reside in close-knit communities, were familiar with the participants' addresses. If a participant relocated during the follow-up period, the date of relocation that was determined based on information from knowledgeable individuals was used as the cutoff date. If the date of relocation was unknown, the last follow-up date was used to censor individuals.

Second survey (KHCS II)

In addition to the follow-up disease registry, a second health survey has been planned for 2023/2024 with the same participants to collect information on anthropometry, biochemical

Table 3. Definitions of severe health outcomes in the Khánh Hòa cardiovascular study, Vietnam (2019–2030).

Health outcomes	Specific diseases listed in the International Classification of Diseases 10th revision (ICD-10)
Mortality	N/A
Cardiovascular diseases	• Ischemic heart diseases except angina pectoris (I21–I25)<br>•Cardiac arrest (I46)<br>•Atrial fibrillation and heart failure (I48–I50)<br>•Cerebrovascular diseases (I60–I69)<br>•Aortic aneurysm and dissection, and other aneurysms (I71–I72)
Neoplasm	• All cancers (malignant neoplasm: C00–C96)[a]<br>•In situ neoplasm / Benign neoplasms / Neoplasms of uncertain or unknown behavior (D00–D48)

ICD: International Classification of Disease.

[a]Information on the other forms of neoplasms (D00–D48) were considered.

https://doi.org/10.1371/journal.pone.0312525.t003

measurements, and questionnaires. This follow-up study would allow us to identify the incidence of minor diseases that cannot be comprehensively calculated using data extracted from the disease registry (e.g., hypertension, diabetes, and depressive symptoms).

Results

Baseline information about the cohort

Information collected from the baseline survey is presented in Table 4. Female participants accounted for 61.3% of the cohort. The mean age (standard deviation [SD]) was 49.9 [5.5] years, and was similar among the sexes (men: 50.2 [SD 5.5] years; women: 49.7 [SD 5.5] years). Almost 90% of the participants were married, and approximately 60% of the participants had attained at least a junior high school education (equivalent to 9 years of formal education), with a higher proportion of those among men (68.5%) than women (53.9%). The proportion of farmers/fishers was higher among men (40.5%) compared with women (21.7%). Approximately four-fifths of the participants were insured (78.3%).

We found that 20.5% of all participants were current smokers, although the majority were men, which indicated a higher smoking prevalence in men (52.2%) than in women (0.4%). Similarly, men reported drinking alcohol more than women (72.1% vs. 2.7%). The median daily cooked rice consumption was 840 g (interquartile range [IQR] = 630–1260 g), with higher consumption among men (median = 1260 g) than in women (median = 630 g). Most participants did not drink coffee or green tea.

The mean [SD] of BMI was 23.2 kg/m^2 [3.0] and the corresponding WHtR was 0.520 [0.054]. The prevalence of hypertension was 39.6% in the study cohort, with a higher prevalence in men (48.1%) than in women (34.3%). Approximately 10% of the participants had diabetes while the corresponding figure for prediabetes was 46.7%. Dyslipidemia was observed in nearly half of the participants (45.1%). After excluding those with CRP $\geq$10 mg/L, who were judged to have acute inflammation (n = 58), 13.0% of the participants had elevated CRP ($\geq$3 mg/L) levels.

Analyses using the baseline information

Using the baseline data, we examined the following associations: parental absence during childhood with depressive symptoms [25], metabolic syndrome [26], and body weight categories (i.e., excess body weight and underweight) [27]; social capital with depressive symptoms [28]; meat consumption with diabetes [29]; tea consumption with diabetes [30]; eating speed with abdominal obesity [31]; physical activity with hypertension [32]; and socioeconomic status with antihypertensive medication among those with hypertension [33].

Disease registry

As of March 2023, 21 participants were recorded in our study-specific registry as deceased, including 5 CVD-related and 12 cancer-related deaths. Furthermore, we registered 22 and 31 cases of nonfatal CVD and cancer events, respectively. Six participants left the study area and were lost to followup. Table 5 presents a comprehensive list of the health events registered in our study.

Discussion

In this article, we describe the data collected in the baseline survey of the KHCS–a prospective cohort study aimed at exploring CVD determinants in the Vietnamese context. We discovered that our study participants were highly burdened with cardiometabolic risk factors, which

Table 4. Summary of selected variables collected in the baseline survey of the Khánh Hòa cardiovascular study, Vietnam (2019–2020), shown by sex.

Variables		All participants (n = 3000)	Male (n = 1160)	Female (n = 1840)
Sociodemographic				
Age	in years, mean [SD]	49.9 [5.5]	50.2 [5.5]	49.7[5.5]
Marital status	Currently married, n (%)	2691 (89.7)	1114 (96.0)	1577 (85.7)
Education	Secondary school or higher, n (%)	1785 (59.5)	794 (68.4)	991 (53.9)
Occupation	Farmer/fisherman, n (%)	870 (29.0)	470 (40.5)	400 (21.7)
Household income	in 10,000 VND, median (IQR)[a]	422.1 (264.6–577.4)	447.2 (288.7–626.1)	404.1 (250–577.4)
Lifestyle-related				
Smoking status	Current smokers, n (%)	614 (20.5)	606 (52.2)	8 (0.4)
Alcohol	Current drinkers, n (%)	886 (29.5)	836 (72.1)	50 (2.7)
Physical activity	Total METs·min/week, median (IQR)	8400 (3980–13440)	9600 (5040–13440)	7840 (3360–13200)
Sleeping hours	<7 hours, n (%)[b]	657 (21.9)	197 (17.0)	460 (25.0)
Rice consumption	g, median (IQR)	840 (630–1260)	1260 (840–1260)	630 (420–840)
Noodle	g, median (IQR)	81.4 (27.1–135.7)	81.4 (54.3–135.7)	81.4 (27.1–135.7)
Bread	Pieces, median (IQR)	0 (0–2)	0 (0–2)	1 (0–2)
Meat consumption	g, median (IQR)	82.9 (42.9–142.9)	108.6 (57.1–194.3)	68.6 (35.7–117.1)
Vegetable	Servings, median (IQR)[c]	1 (1–2)	1 (1–2)	1 (1–2)
Fruit	Servings, median (IQR)[c]	0.5 (0.21–1.00)	0.43 (0.14–1.00)	0.5 (0.29–1.00)
Sweetened beverage	Cans, median (IQR)	0.0 (0.0–0.0)	0.0 (0.0–0.0)	0.0 (0.0–0.0)
Coffee	Cups, median (IQR)[d]	0.0 (0.0–0.86)	0.43 (0.0–1.0)	0.0 (0.0–0.3)
Green tea	Cups, median (IQR)[e]	0.0 (0.0–0.14)	0.0 (0.0–3.0)	0.0 (0.0–0.0)
Social environment				
Social capital	Social participation, n (%)	1125 (37.5)	405 (34.9)	720 (39.1)
Childhood SES	Low SES at age 15, n (%)	1947 (64.9)	738 (63.6)	1209 (65.7)
Parental absence	Parental absence before age 15, n (%)	636 (21.2)	235 (20.3)	401 (21.8)
Health-related				
Body mass index	Mean [SD]	23.2 [3.0]	22.9 [3.1]	23.4 [2.9]
Waist-to-height ratio	Mean [SD]	0.520 [0.054]	0.510 [0.053]	0.526 [0.053]
Excess body weight	BMI $\geq$25.0 kg/m^2, n (%)	778 (25.9)	285 (24.6)	493 (26.8)
Hypertension	n (%)	1189 (39.6)	558 (48.1)	631 (34.3)
Systolic blood pressure	Mean [SD]	130.9 [19.1]	133.8 [19.3]	129.1 [18.7]
Diastolic blood pressure	Mean [SD]	83.9 [12.1]	87.4 [12.5]	81.7 [11.4]
Diabetes	n (%)	307 (10.2)	122 (10.5)	185 (10.1)
Prediabetes	n (%)	1401 (46.7)	565 (48.7)	836 (45.4)
Fasting plasma glucose	Mean [SD]	99.9 [26.7]	101.4 [26.0]	99.0 [27.1]
HbA1c	Mean [SD]	5.8 [1.0]	5.8 [0.9]	5.8 [1.0]
Dyslipidemia	n (%)	1352 (45.1)	592 (51.0)	760 (41.3)
Total cholesterol	Mean [SD]	198.7 [40.3]	194.4 [40.1]	201.5 [40.1]
Triglycerides	Mean [SD]	174.0 [133.7]	212.5 [176.9]	149.8 [88.8]
Low-density lipoprotein cholesterol	Mean [SD]	126.2 [35.9]	116.9 [34.6]	132.0 [35.4]
High-density lipoprotein cholesterol	Mean [SD]	51.4 [12.9]	49.8 [14.2]	52.3 [11.9]
Metabolic syndrome	n (%)	1151 (38.4)	424 (36.6)	727 (39.5)
Subclinical inflammation	C-reactive protein 3.0–9.9 mg/L, n (%)[6]	381 (13.0)	130 (11.5)	251 (13.9)

(*Continued*)

Table 4. (Continued)

Variables		All participants (n = 3000)	Male (n = 1160)	Female (n = 1840)
**C-reactive protein**	**Mean [SD]**	1.6 [1.5]	1.5 [1.5]	1.6 [1.6]

BMI, body mass index; IQR, interquartile range; MET, metabolic equivalents; SD, standard deviation; VND: Vietnamese Dong.

[a]Household income was estimated based on the responses from a participant who represented the given household. This information was available for 2967 individuals.

[b]The sum of nighttime sleep and naps. The information was available for 2999 individuals (1159 men and 1840 women).

[c]Results are shown in terms of one serving shown in a show card (125 mL or 1/2 cup).

[d]Results are shown in terms of one serving depicted in a show card (40 mL)

[e]Results are shown in terms of one serving depicted in a show card (65 mL).

[f]Participants with CRP ≥10 mg/L, defined as acute inflammation, and were excluded; thus, 2942 participants (1130 men and 1812 women) were included.

https://doi.org/10.1371/journal.pone.0312525.t004

conferred a high risk for CVD or other major NCDs. The majority of those who smoked and consumed alcohol were men, whereas these unhealthy behaviors were uncommon among women, which should be considered in subsequent research.

The WHO STEP survey [34], which was conducted among individuals aged 18–69 years in all 63 provinces and cities of Vietnam, reported a similar prevalence of hypertension and excess body weight as in our study. Although the age categories used in the report to summarize the findings do not match the age group of the present study cohort, when we used the publicly available STEP Survey data for comparison and calculated the prevalence for the same age category (i.e., 40–60 years), the prevalences of hypertension were 35.6% and 25.0% and of excess body weight (BMI ≥25 kg/m^2) were 17.2% and 23.9% among men and women,

Table 5. Fatal and non-fatal health events registered in the Khánh Hòa cardiovascular study as of March 2023.

Health events	ICD-10 code	Specific diseases	Fatal cases, n	Non-fatal cases, n
**CVD**	**I21–I25**	Ischemic heart diseases except angina pectoris	1	9
	**I46**	Cardiac arrest	0	0
	**I48–I50**	Atrial fibrillation and heart failure	0	2
	**I60–I69**	Cerebrovascular diseases	4	11
	**I71–I72**	Aortic aneurysm and dissection and other aneurysms	0	0
	-	Subtotal	5	22
**Neoplasm**	**C00–C14**	Malignant neoplasms of lip, oral cavity and pharynx	0	1
	**C15–C26**	Malignant neoplasms of digestive organs	7	9
	**C30–C39**	Malignant neoplasms of respiratory and intrathoracic organs	2	3
	**C45–C49**	Malignant neoplasms of mesothelial and soft tissue	1	1
	**C50**	Malignant neoplasm of breast	1	7
	**C51–C58**	Malignant neoplasms of female genital organs	0	1
	**C69–C72**	Malignant neoplasms of eye, brain and other parts of central nervous system	0	1
	**C73–C75**	Malignant neoplasms of thyroid and other endocrine glands	0	6
	**C81–C96**	Malignant neoplasms, stated or presumed to be primary, of lymphoid, haematopoietic and related tissue	1	2
	**D00–D48**	In situ neoplasm / Benign neoplasms / Neoplasms of uncertain or unknown behaviour	1	7
	-	Subtotal	13	38
**Other causes**	**A41**	Sepsis	1	-
	-	Unknown	2	-
-	-	Total	21	61

CVD: Cardiovascular diseases; ICD-10: International Classification of Diseases 10th Revision.

https://doi.org/10.1371/journal.pone.0312525.t005

respectively. Although these values were lower than those reported in our survey, these comparative results suggest that our study population and the general population in Vietnam have a high cardiovascular risk (S2 Table). Thus, the evidence from this study group could potentially contribute to improved public health initiatives in Vietnam.

Furthermore, our baseline results also suggest that there are also several concerning aspects as regards health-related lifestyle habits, particularly among men. For example, the prevalence of smoking among males was particularly high, with the prevalence of smoking being 52.2%, which was equivalent to the figure reported in a study using the STEP survey questionnaire in Vietnam [35]. Given that smoking has health effects [36,37], the high smoking rate in Vietnam suggests the possibility of various health problems arising from both active and secondhand smoke. Quitting smoking can reduce the risk of several health outcomes [38–40], and thus, necessary measures should be taken. In addition, the prevalence of current alcohol drinkers among men was high (72.1%), which warrants attention because the evidence has increasingly shown a lack of support for a beneficial health effect of low- to moderate-alcohol consumption [41,42].

Using cross-sectional data collected from the baseline survey, we have identified similarities and differences in the determinants of health between Vietnam and countries where prior studies have been conducted. Our finding that meat consumption is positively associated with diabetes [43,44] and that individuals with higher social capital exhibit fewer depressive symptoms [45,46] are consistent with previously reported results. However, the positive association found between green tea consumption and diabetes observed in our dataset [30] differed from previous studies that reported inverse associations [47,48]. Regarding physical activity and hypertension, our study reported an inverse association with occupational physical activity but not with leisure-time physical activity, which has often been linked with a reduction in hypertension risk in previous studies [49,50]. Additionally, when we examined the association with parental absence, we found a significant association with low body weight but not with metabolic syndrome, although adverse childhood experiences in general have been suggested to result in metabolic abnormalities [51]. These findings suggest the need for epidemiological research that accounts for possible differences in the effects of health determinants.

Currently, we do not have a sufficient number of negative health events for the survival analysis. Therefore, we plan to conduct a longitudinal survival analysis after a maximum follow-up period of 10 years. Moreover, we are scheduling the second round of the KHCS survey inviting the same individuals in 2023 and 2024. During this, we intend to collect blood samples and questionnaire data again. As the disease registration system is unsuitable for capturing the onset of less severe health conditions (e.g., diabetes, hypertension, and depressive symptoms) in locations with limited access to medical services, we plan to define the onset of these conditions using data collected in the second wave of the study. Additionally, the research topics examined earlier using cross-sectional data will be re-evaluated longitudinally. Accordingly, we aim to produce high-quality epidemiological evidence that reflects the Vietnamese context.

This study has several strengths, including its focus on a rural population in a low-to-middle-income country, which enhances its contribution to epidemiological research by providing valuable insights into health outcomes in underrepresented and underserved communities. However, this study also has several limitations. First, as participants of this study were not selected via a random sampling procedure and did not fully represent the general Vietnamese population, or even inhabitants of the Khánh Hòa Province, caution should be exercised when generalizing the findings to other populations. Heterogeneity in the characteristics across the study communes may exist, which should be accounted for when conducting analyses. For example, using multilevel models can help accommodate variations within and between communes. Second, this study was observational in nature, and exposures were not randomly

assigned; factors determining exposures might influence health outcomes (i.e., unmeasured confounding). Third, some exposure information was self-reported, which introduced the possibility of recall and social desirability biases. Fourth, although we used a validated questionnaire to measure physical activity, the estimates were deemed quite high, which might have resulted from exaggerated responses. A similar limitation was reported in a previous study that examined the reliability of the GPAQ in Vietnam, which highlighted that estimates from Vietnam were particularly exaggerated among those who lived in rural areas [52]. Fifth, the results of the cross-sectional analysis cannot deny the possibility of reverse causation; therefore, caution is required when interpreting the findings. Future longitudinal studies should address these limitations. Sixth, although the disease registry system combines not only local medical databases but also telephonic confirmation with individuals, family members, and household visits to ensure that no case was missed, we cannot rule out the possibility that the capture rate might be lower than expected in HICs.

Conclusions

The KHCS is a prospective cohort study among 3000 rural residents in Khánh Hòa Province, Vietnam. We conducted a baseline survey to collect information on anthropometry, biochemical measurements, lifestyle parameters, and social determinants of health. With the outcome information collected in the disease registry and the second health survey (KHCS II), this study group will produce robust evidence that would facilitate the effort to combat the CVD- and NCD-associated disease burden in Vietnam.

Supporting information

S1 Table. Specific criteria used to define health outcomes in the Khánh Hòa cardiovascular study, Vietnam.
(DOCX)

S2 Table. Prevalence of selected health-related outcomes ascertained from the STEP survey data.
(DOCX)

Acknowledgments

We are grateful to the participants of the Khánh Hòa Cardiovascular Study for their participation.

Author Contributions

Conceptualization: Danh Cong Phan, Hung Thai Do, Tetsuya Mizoue, Yosuke Inoue.

Data curation: Chau Que Nguyen, Thuy Thi Phuong Pham.

Formal analysis: Chau Que Nguyen, Yosuke Inoue.

Funding acquisition: Yosuke Inoue.

Methodology: Chau Que Nguyen, Yosuke Inoue.

Supervision: Hung Thai Do, Tetsuya Mizoue, Yosuke Inoue.

Writing – original draft: Chau Que Nguyen, Thuy Thi Phuong Pham, Yosuke Inoue.

Writing – review & editing: Danh Cong Phan, Hung Thai Do, Tetsuya Mizoue.

References

1. GBD 2019 Diseases and Injuries Collaborators. Global burden of 369 diseases and injuries in 204 countries and territories, 1990–2019: a systematic analysis for the Global Burden of Disease Study 2019. Lancet. 2020; 396:1204–22. https://doi.org/10.1016/S0140-6736(20)30925-9 PMID: 33069326
2. Roth GA, Mensah GA, Johnson CO, Addolorato G, Ammirati E, Baddour LM, et al. Global Burden of Cardiovascular Diseases and Risk Factors, 1990–2019: Update From the GBD 2019 Study. J Am Coll Cardiol. 2020; 76:2982–3021. https://doi.org/10.1016/j.jacc.2020.11.010 PMID: 33309175
3. Tsao CW, Aday AW, Almarzooq ZI, Anderson CAM, Arora P, Avery CL, et al. Heart Disease and Stroke Statistics 2023 Update: A Report From the American Heart Association. Circulation. 2023; 147: e93–621. https://doi.org/10.1161/CIR.0000000000001123 PMID: 36695182
4. Powell-Wiley TM, Baumer Y, Baah FO, Baez AS, Farmer N, Mahlobo CT, et al. Social Determinants of Cardiovascular Disease. Circulation Research. 2022; 130:782–99. https://doi.org/10.1161/CIRCRESAHA.121.319811 PMID: 35239404
5. Franzen SRP, Chandler C, Lang T. Health research capacity development in low- and middle-income countries: reality or rhetoric? A systematic meta-narrative review of the qualitative literature. BMJ Open. 2017; 7: e012332. https://doi.org/10.1136/bmjopen-2016-012332 PMID: 28131997
6. Anand S, Bradshaw C, Prabhakaran D. Prevention and management of CVD in LMICs: why do ethnicity, culture, and context matter? BMC Medicine. 2020; 18:7. https://doi.org/10.1186/s12916-019-1480-9 PMID: 31973762
7. Bowry ADK, Lewey J, Dugani SB, Choudhry NK. The Burden of Cardiovascular Disease in Low- and Middle-Income Countries: Epidemiology and Management. Can J Cardiol. 2015; 31: 1151–9. https://doi.org/10.1016/j.cjca.2015.06.028 PMID: 26321437
8. The Government of the Socialist Republic of Vietnam. Vietnam history overview. http://vietnam.gov.vn/history-68964. Accessed 23 Oct 2023.
9. International Monetary Fund. World Economic Outlook (October 2023). https://www.imf.org/external/datamapper/datasets/WEO. Accessed 3 Nov 2023.
10. United Nations. World Population Prospects 2022—Viet Nam. https://population.un.org/wpp/Graphs/DemographicProfiles/Line/704. Accessed 23 Oct 2023.
11. The Institute for Health Metrics and Evaluation. Vietnam. https://www.healthdata.org/research-analysis/health-by-location/profiles/vietnam. Accessed 22 Oct 2023.
12. Nha Trang climate: Average Temperature by month, Nha Trang water temperature. https://en.climate-data.org/asia/vietnam/khanh-hoa-province/nha-trang-4270/. Accessed 18 Feb 2024.
13. World Health Organization. STEPwise approach to NCD risk factor surveillance (STEPS). https://www.who.int/teams/noncommunicable-diseases/surveillance/systems-tools/steps. Accessed 23 Oct 2023.
14. Tsubono Y, Kobayashi M, Sasaki S, Tsugane S, JPHC. Validity and reproducibility of a self-administered food frequency questionnaire used in the baseline survey of the JPHC Study Cohort I. J Epidemiol. 2003; 13 1 Suppl: S125–133.
15. Emmett PM, Jones LR, Northstone K, Pounis G, Taylor CM. Chapter 3—Collection and Management of Dietary Data. In: Pounis G, editor. Analysis in Nutrition Research. Academic Press; 2019. p. 43–73.
16. Tran T, Harpham T, Huong N, De Silva M, Huong V, Long T, et al. Validity of a Social Capital Measurement Tool in Vietnam. Asian Journal of Social Science. 2005; 33:208–22.
17. Kohout FJ, Berkman LF, Evans DA, Cornoni-Huntley J. Two Shorter Forms of the CES-D Depression Symptoms Index. Journal of Aging and Health. 1993; 5:179–93.
18. Carmienke S, Freitag MH, Pischon T, Schlattmann P, Fankhaenel T, Goebel H, et al. General and abdominal obesity parameters and their combination in relation to mortality: a systematic review and meta-regression analysis. Eur J Clin Nutr. 2013; 67:573–85. https://doi.org/10.1038/ejcn.2013.61 PMID: 23511854
19. Abdi Dezfouli R, Mohammadian Khonsari N, Hosseinpour A, Asadi S, Ejtahed H-S, Qorbani M. Waist to height ratio as a simple tool for predicting mortality: a systematic review and meta-analysis. Int J Obes. 2023; 47:1286–301. https://doi.org/10.1038/s41366-023-01388-0 PMID: 37770574
20. World Health Organization. A healthy lifestyle—WHO recommendations. https://www.who.int/europe/news-room/fact-sheets/item/a-healthy-lifestyle—who-recommendations. Accessed 24 Jan 2024.
21. World Health Organization. Hypertension. https://www.who.int/news-room/fact-sheets/detail/hypertension. Accessed 27 Jan 2024.
22. American Diabetes Association. 2. Classification and Diagnosis of Diabetes: Standards of Medical Care in Diabetes—2020. Diabetes Care. 2020; 43 Supplement 1: S14–31.
23. Expert Panel on Detection, Evaluation, and Treatment of High Blood Cholesterol in Adults. Executive Summary of the Third Report of the National Cholesterol Education Program (NCEP) Expert Panel on

Detection, Evaluation, and Treatment of High Blood Cholesterol in Adults (Adult Treatment Panel III). JAMA. 2001; 285:2486–97. https://doi.org/10.1001/jama.285.19.2486 PMID: 11368702

24. Pearson TA, Mensah GA, Hong Y, Smith SC. CDC/AHA Workshop on Markers of Inflammation and Cardiovascular Disease: Application to Clinical and Public Health Practice: Overview. Circulation. 2004; 110: e543–544. https://doi.org/10.1161/01.CIR.0000148979.11121.6B PMID: 15611378

25. Inoue Y, Fukunaga A, Stickley A, Yazawa A, Pham TTP, Nguyen CQ, et al. Association between parental absence during childhood and depressive symptoms in adulthood in rural Vietnam. J Affect Disord. 2022; 311:479–85. https://doi.org/10.1016/j.jad.2022.05.102 PMID: 35598745

26. Shrestha RM, Mizoue T, Pham TTP, Fukunaga A, Hoang DV, Nguyen CQ, et al. Association between parental absence during childhood and metabolic syndrome during adulthood: A cross-sectional study in rural Khanh Hoa, Vietnam. PLOS ONE. 2023; 18: e0282731. https://doi.org/10.1371/journal.pone.0282731 PMID: 36893153

27. Yazawa A, Kawachi I, Shrestha RM, Fukunaga A, Pham TTP, Nguyen CQ, et al. Parental absence during childhood and weight status in adulthood among middle-aged community dwellers in rural Vietnam. American Journal of Human Biology. 2023; 35: e23827. https://doi.org/10.1002/ajhb.23827 PMID: 36345160

28. Do AD, Pham TTP, Nguyen CQ, Hoang DV, Fukunaga A, Stickley A, et al. Individual-level social capital is associated with depressive symptoms among middle-aged community dwellers in rural Vietnam: a cross-sectional study. BMJ Open. 2022; 12: e064998. https://doi.org/10.1136/bmjopen-2022-064998 PMID: 36600355

29. Nguyen CQ, Pham TTP, Fukunaga A, Hoang DV, Phan TV, Phan DC, et al. Red meat consumption is associated with prediabetes and diabetes in rural Vietnam: a cross-sectional study. Public Health Nutr. 2023; 26:1006–13. https://doi.org/10.1017/S1368980022001422 PMID: 35722988

30. Fukunaga A, Jimba M, Pham TTP, Nguyen CQ, Hoang DV, Phan TV, et al. Association of green tea consumption with prediabetes, diabetes and markers of glucose metabolism in rural Vietnam: a cross-sectional study. British Journal of Nutrition. 2024; 131:1883–91. https://doi.org/10.1017/S0007114524000412 PMID: 38361457

31. Hoang DV, Fukunaga A, Nguyen CQ, Pham TTP, Shrestha RM, Phan DC, et al. Eating speed and abdominal adiposity in middle-aged adults: a cross-sectional study in Vietnam. BMC Public Health. 2023; 23:443. https://doi.org/10.1186/s12889-023-15328-0 PMID: 36882714

32. Do AD, Pham TTP, Nguyen CQ, Van Hoang D, Fukunaga A, Yamamoto S, et al. Different associations of occupational and leisure-time physical activity with the prevalence of hypertension among middle-aged community dwellers in rural Khánh Hòa, Vietnam. BMC Public Health. 2023; 23:713.

33. Yokobori Y, Fukunaga A, Okawa S, Hachiya M, Nguyen CQ, Pham TPT, et al. Sex differences in the association between socioeconomic status and untreated hypertension among residents with hypertension in rural Khánh Hòa, Vietnam: a post-hoc analysis. BMC Cardiovascular Disorders. 2024; 24:61.

34. World Health Organization. 2015 STEPS Country report Viet Nam. https://www.who.int/publications/m/item/2015-steps-country-report-viet-nam. Accessed 5 Nov 2023.

35. Bui TV, Blizzard L, Luong KN, Truong NLV, Tran BQ, Ha ST, et al. Declining Prevalence of Tobacco Smoking in Vietnam. Nicotine & Tobacco Research. 2015; 17:831–8. https://doi.org/10.1093/ntr/ntu202 PMID: 25332457

36. World Health Organization. Tobacco. https://www.who.int/news-room/fact-sheets/detail/tobacco. Accessed 2 Dec 2023.

37. United States Centers for Disease Control and Prevention. Health Effects of Cigarette Smoking. Centers for Disease Control and Prevention. 2022. https://www.cdc.gov/tobacco/data_statistics/fact_sheets/health_effects/effects_cig_smoking/index.htm. Accessed 2 Dec 2023.

38. Chang JT, Anic GM, Rostron BL, Tanwar M, Chang CM. Cigarette Smoking Reduction and Health Risks: A Systematic Review and Meta-analysis. Nicotine Tob Res. 2021; 23:635–42. https://doi.org/10.1093/ntr/ntaa156 PMID: 32803250

39. Pan A, Wang Y, Talaei M, Hu FB, Wu T. Relation of active, passive, and quitting smoking with incident type 2 diabetes: a systematic review and meta-analysis. Lancet Diabetes Endocrinol. 2015; 3:958–67. https://doi.org/10.1016/S2213-8587(15)00316-2 PMID: 26388413

40. Taylor G, McNeill A, Girling A, Farley A, Lindson-Hawley N, Aveyard P. Change in mental health after smoking cessation: systematic review and meta-analysis. BMJ. 2014; 348: g1151. https://doi.org/10.1136/bmj.g1151 PMID: 24524926

41. Griswold MG, Fullman N, Hawley C, Arian N, Zimsen SRM, Tymeson HD, et al. Alcohol use and burden for 195 countries and territories, 1990–2016: a systematic analysis for the Global Burden of Disease Study 2016. The Lancet. 2018; 392:1015–35. https://doi.org/10.1016/S0140-6736(18)31310-2 PMID: 30146330

42. Zhao J, Stockwell T, Naimi T, Churchill S, Clay J, Sherk A. Association Between Daily Alcohol Intake and Risk of All-Cause Mortality: A Systematic Review and Meta-analyses. JAMA Network Open. 2023; 6: e236185. https://doi.org/10.1001/jamanetworkopen.2023.6185 PMID: 37000449

43. Aune D, Ursin G, Veierød MB. Meat consumption and the risk of type 2 diabetes: a systematic review and meta-analysis of cohort studies. Diabetologia. 2009; 52:2277–87. https://doi.org/10.1007/s00125-009-1481-x PMID: 19662376

44. Pan A, Sun Q, Bernstein AM, Schulze MB, Manson JE, Willett WC, et al. Red meat consumption and risk of type 2 diabetes: 3 cohorts of US adults and an updated meta-analysis. The American Journal of Clinical Nutrition. 2011; 94:1088–96. https://doi.org/10.3945/ajcn.111.018978 PMID: 21831992

45. De Silva MJ, McKenzie K, Harpham T, Huttly SRA. Social capital and mental illness: a systematic review. Journal of Epidemiology and Community Health. 2005; 59:619–27. https://doi.org/10.1136/jech.2004.029678 PMID: 16020636

46. Ehsan AM, De Silva MJ. Social capital and common mental disorder: a systematic review. J Epidemiol Community Health. 2015; 69:1021–8. https://doi.org/10.1136/jech-2015-205868 PMID: 26179447

47. Huang H, Guo Q, Qiu C, Huang B, Fu X, Yao J, et al. Associations of Green Tea and Rock Tea Consumption with Risk of Impaired Fasting Glucose and Impaired Glucose Tolerance in Chinese Men and Women. PLOS ONE. 2013; 8: e79214. https://doi.org/10.1371/journal.pone.0079214 PMID: 24260170

48. Iso H, Date C, Wakai K, Fukui M, Tamakoshi A, JACC Study Group. The relationship between green tea and total caffeine intake and risk for self-reported type 2 diabetes among Japanese adults. Ann Intern Med. 2006; 144:554–62.

49. Huai P, Xun H, Reilly KH, Wang Y, Ma W, Xi B. Physical Activity and Risk of Hypertension. Hypertension. 2013; 62:1021–6.

50. Liu X, Zhang D, Liu Y, Sun X, Han C, Wang B, et al. Dose–Response Association Between Physical Activity and Incident Hypertension. Hypertension. 2017; 69:813–20.

51. Danese A, Moffitt TE, Harrington H, Milne BJ, Polanczyk G, Pariante CM, et al. Adverse Childhood Experiences and Adult Risk Factors for Age-Related Disease: Depression, Inflammation, and Clustering of Metabolic Risk Markers. Archives of Pediatrics & Adolescent Medicine. 2009; 163:1135–43. https://doi.org/10.1001/archpediatrics.2009.214 PMID: 19996051

52. Bui TV, Blizzard CL, Luong KN, Truong NLV, Tran BQ, Otahal P, et al. Physical Activity in Vietnam: Estimates and Measurement Issues. PLOS ONE. 2015; 10: e0140941. https://doi.org/10.1371/journal.pone.0140941 PMID: 26485044
